# Chemical composition, antioxidant, and antimicrobial properties of *Mentha subtomentella*: in sight *in vitro* and *in silico* analysis

**DOI:** 10.3389/fchem.2023.1341704

**Published:** 2024-01-19

**Authors:** Fatima Brahmi, Nour Eddine Bentouhami, Youssef Rbah, Amine Elbouzidi, Ouafae Mokhtari, Ahmad Mohammad Salamatullah, Samir Ibenmoussa, Mohammed Bourhia, Mohamed Addi, Abdeslam Asehraou, Bouchra Legssyer

**Affiliations:** ^1^ Laboratory for the Improvement of Agricultural Production, Biotechnology, and Environment, University Mohammed Premier, Oujda, Morocco; ^2^ Laboratory of Bioresources, Biotechnology, Ethnopharmacology and Health, Faculty of Science, University Mohammed Premier, Oujda, Morocco; ^3^ Euromed University of Fez, Fez, Morocco; ^4^ Department of Food Science and Nutrition, College of Food and Agricultural Sciences, King Saud University, Riyadh, Saudi Arabia; ^5^ Laboratory of Therapeutic and Organic Chemistry, Faculty of Pharmacy, University of Montpellier, Montpellier, France; ^6^ Department of Chemistry and Biochemistry, Faculty of Medicine and Pharmacy, Ibn Zohr University, Laayoune, Morocco; ^7^ Laboratory of Chemistry-Biochemistry, Environment, Nutrition, and Health, Faculty of Medicine and Pharmacy, Casablanca, Morocco

**Keywords:** M. subtomentella, essential oil, aqueous extract, GC/MS, HPLC/DAD, antimicrobial activity, antioxidant activity, molecular docking

## Abstract

Our research focused on assessing essential oils (MSEO) and aqueous extracts (MSAE) derived from M. subtomentella leaves, with a primary focus on evaluating their properties. From 1 kg of leaves, we successfully obtained 18 mL of essential oil. Upon conducting GC/MS analysis, we identified eleven compounds within the oil, collectively accounting for 100% of the constituents identified. Notably, the predominant compounds in the leaf oil were p-Menth-48) -en-3-one (50.48%), 9-Ethylbicyclo (3.3.1) nonan-9-ol (10.04%) (E)-3,3-Dimethyl-delta-1, alpha-cyclohexaneacetaldehyde (8.53%), and D-Limonene (7.22%). Furthermore, utilizing HPLC/DAD, we explored the phenolic profile of MSAE, extracted through decoction. This analysis revealed the presence of fifty-eight compounds, with five major components collectively constituting 61% of the total compounds identified, rosmarinic acid as the major one. We evaluated the antimicrobial effectiveness of the MSEO against ten different strains, observing its notable efficacy against *A. Niger* (MIC = 0.09%), *P. digitatum* (MIC = 0.5%), and *G. candidum* (MIC = 1%). However, the essential oil demonstrated comparatively lower efficacy against bacteria than fungi. In contrast, the MSAE did not exhibit any antimicrobial activity against the tested strains. Regarding antioxidant activity, the aqueous extract displayed a significantly higher antioxidant capacity than the essential oil, which exhibited relatively lower antioxidant activity. The IC_50_ values were determined to be 0.04 ± 0.01 mg/mL, 0.17 ± 0.01 mg/mL, and 13% ± 0.01% (V/V), for ascorbic acid MSAE and MSEO, respectively. We used a computational method called molecular docking to investigate how certain plant compounds affect antioxidant, antibacterial, and antifungal activities. This involved analyzing the interactions between these compounds and specific protein targets known for their roles in these activities.

## Introduction

The geographical diversity of Morocco has fostered an extraordinary botanical wealth. The country’s Mediterranean bioclimates, ranging from the Mediterranean coast to the Sahara Desert, have created an environment conducive to the evolution of a vast and unique plant species. This biodiversity extends to over 4,800 documented plant species, making Morocco’s botanical mosaic rich in endemism and utility (Fennane and Mohamed, 2018).

Among the botanical treasures concealed within Morocco’s landscapes, the Mentha genus, in particular, is prominent. Within this genus, Mentha subtomentella var. humillima (M. subtomentella), known locally as flio, is a testament to nature’s ingenuity. With an estimated 25 distinct species and hybrids, this aromatic herb has been integral to Morocco’s cultural and medicinal heritage for generations (Fennane and Mohamed, 2018).

The applications of M. subtomentella in Moroccan traditional medicine are extensive and diverse (Jamila and Mostafa, 2014). Its reputation as a potent antispasmodic precedes it, and it has been employed to alleviate the discomfort of conditions such as colds, bronchitis, and sinusitis (Najem et al., 2021). M. subtomentella’s utility extends further into gastrointestinal health, relieving cholera, food poisoning, flatulence, and intestinal colic inflammation. It is also cherished for its soothing effects on the skin, making it a go-to remedy for dermatological discomfort (Ajjoun et al., 2022). Beyond this, it has been utilized to combat gastric and intestinal pain and address helminthiases, underscoring its versatility within herbal medicine.

Previous studies have shown promising results in exploring the pharmacological potential of M. subtomentella. This plant extract has demonstrated antibacterial and antifungal activities, making it a natural alternative for addressing microbial threats (Abbou et al., 2022). Additionally, it exhibits anticholinesterase and anti-inflammatory effects that can relieve many ailments (Benabdallah et al., 2018; Yakoubi et al., 2021). The essential oil derived from M. subtomentella has remarkable properties, including anthelmintic and anticancer effects, highlighting its potential for diverse medical applications (Alami Merrouni and Elachouri, 2021; Hashemi et al., 2023). The intricate chemical composition of M. subtomentella includes flavonoids, benzyl and hydroisocoumarins, phenolic compounds, terpenes, saponins, and sterols (Gulcin, 2020; Khetabi et al., 2023). Many of these chemical constituents play a role in the plant’s therapeutic properties and classification within the botanical kingdom.

The main purpose of this research is to explore the different properties of M. subtomentella. The study aims to assess the antioxidant and antimicrobial potential *in vitro* and *in silico* of the essential oil and aqueous extracts obtained from the plant’s aerial parts, as well as their composition. By delving into these aspects in more detail, this research seeks to reveal the complete range of therapeutic and chemical attributes of M. subtomentella. This could result in increased utilization of this plant in pharmaceutical, agricultural, and other industries.

## Results and discussion

### Chemical composition

**FIGURE 1:**
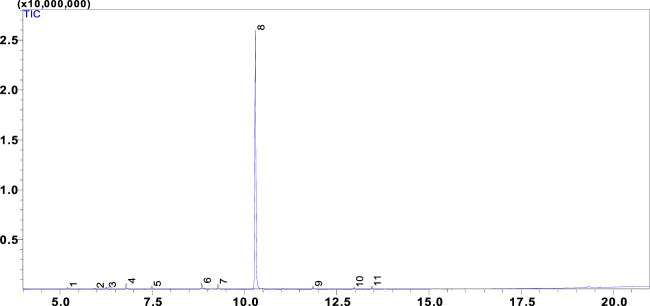
Chromatogram of MSEO.

1 kg of M. subtomentella Leaves were extracted, and 18 mL of essential oil was obtained. The general chemical profile of the tested oil, the percentage content of the individual components, the retention indices, and the chemical class distribution of the oil compounds are summarized in (Table 1). Eleven components were identified in the tested oil (Figure 1), representing 100% of the total detected constituent. The major components of the leaves were p-Menth-4 (8)-en-3-one (50.48%), 9-Ethylbicyclo (3.3.1) nonan-9-ol (10.04%) (E)-3,3-Dimethyl-. Delta.1, alpha cyclohexane acetaldehyde (8.53%), and D-Limonene (7.22%). Other components were present in amounts less than 5%.

**TABLE 1:** Chemical compounds of essential oil extracted from M. subtomentella.

N°	Compounds	RT	Area (%)
1	α -Pinene	5.209	2.29 ± 0.00
2	β -Pinene	5.938	1.64 ± 0.00
3	3-Octanol	6.247	3.26 ± 0.00
4	D-Limonene	6.793	7.22 ± 0.00
5	p-Mentha-3,8-diene	7.487	4.05 ± 0.00
6	9-Ethylbicyclo (3.3.1)nonan-9-ol	8.841	10.04 ± 0.00
7	(E)-3,3-Dimethyl-.delta.1,.alpha.-cyclohexaneacetaldehyde	9.278	8.53 ± 0.00
8	p-Menth-4 (8)-en-3-one	10.297	50.48 ± 0.00
9	Verbenone	11.856	4.80 ± 0.00
10	Caryophyllene	12.973	3.08 ± 0.00
11	1.5,9,9-Tetramethyl-1,4,7-cycloundecatriene	13.454	4.61 ± 0.00

RT: retention time.

Several studies have investigated the chemical composition of the MSEO is grown in different geographical regions, which reveals a great variability in its chemical profile it has different chemotypes, and oils with high content of pulegone (Ait-Ouazzou et al., 2012) and oils with low levels of pulegone but rich in menthone/iso menthone, or piperitenone/piperine, or piperitone (Mahboubi and Ghasem, 2008; Abdelli et al., 2016). The variation in the essential oil content and composition of M. subtomentella plants were related to a variety of factors, such as genotypes of Mentha species, environmental conditions, season, plant age, and different plant parts (Barzalona and Casanova, 2008).

### HPLC/DAD results

The chemical compounds of the MSAE were characterized using high-performance liquid chromatography coupled to a diode array detector (HPLC-DAD) by analyzing their retention times and absorbance spectra, which were compared to previously published research studies.

**FIGURE 2:**
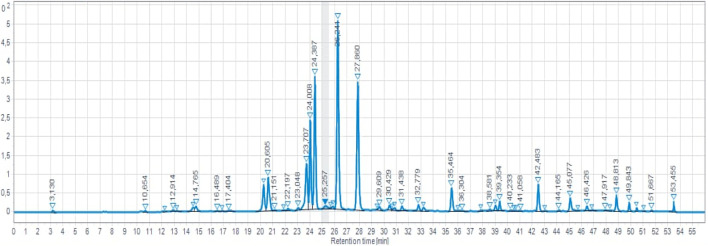
HPLC-DAD chromatogram of MSAE.

A total of 58 compounds were detected within the MSAE, comprising 100% of the total identified compounds as shown in (Figure 2; Table 2). Among these, we successfully identified five major compounds, in comparison to the findings of F. Ferreres in 2015 and R. Vilamarim in 2018, namely, Rosmarinic acid (22.53%), Feruloyl hexosylpentoside (16.21%), Caffeoyl hexosylpentoside (13.94%), Salvianolic acid H (8.74%) (Ferreres et al., 2015). Other studies have revealed the presence of additional major compounds. For instance, in N. K. Alharbi et al. (2021) the following major compounds were identified: Eriocitrin 12.5%, Hesperidin 17.5%, Narirutin 16.25%, Luteolin 15.5%, Isorhoifolin 14%, Rosmarinic acid 18.5%, and Caffeic acid 10.5% (Brown et al., 2019).

**TABLE 2:** Major phenolic compounds of MSAE.

N°	Compounds	RT	Area (%)
1	Salvianolic acid H	24.008	8.741 ± 0.00
2	Caffeoyl hexosylpentoside	24.387	13.942 ± 0.00
3	Rosmarinic acid	26.244	22.533 ± 0.00
4	Feruloyl hexosylpentoside	27.860	16.212 ± 0.00

### Antioxidant activity

**FIGURE 3:**
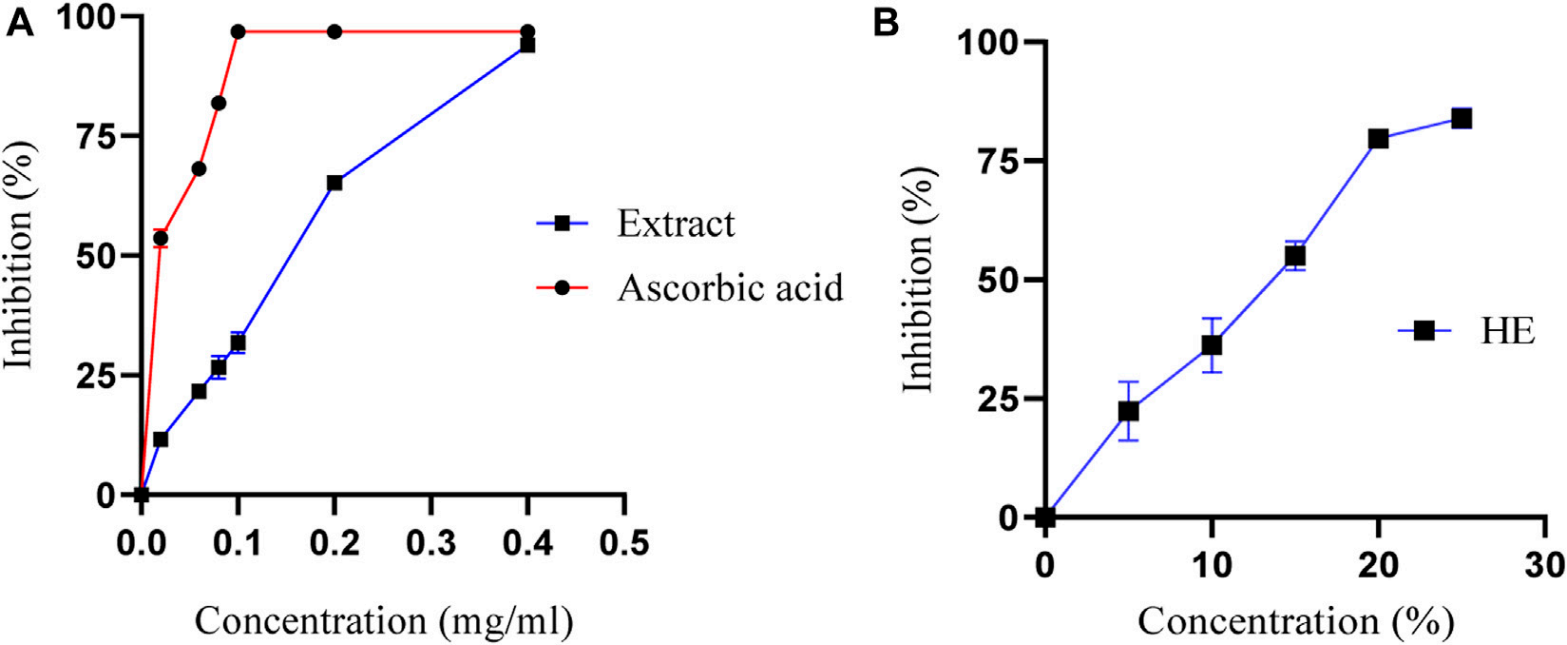
**(A)**The percentage of antioxidant activity of MSAE and ascorbic acid as a function of their concentration **(B)** The percentage of antioxidant activity of MSEO as a function of their concentration.

The antioxidant activity of M. subtomentella extracts is assessed using the DPPH radical (Taslimi and Gulçin, 2018; Durmaz et al., 2022; Yılmaz et al., 2023), which measures the capacity of substances to act as radical scavengers or hydrogen donors, thereby evaluating their antioxidant properties. The DPPH radical remains stable under normal conditions and can accept an electron or hydrogen atom to transform into a stable paramagnetic molecule. The MSEO, and MSAO were evaluated using spectrophotometry at 517 nm to measure the reduction of the DPPH radical, which is accompanied by a color change from purple to yellow. The percentage of antioxidant activity of both essential oil and aqueous extract of M. subtomentella. is shown in Figure 3, with results indicating a significantly high level of antioxidant effect, reaching up to 93.97%, a value close to that of ascorbic acid, which had a maximum value of approximately 96.80%. IC_50_ values were 0.04 ± 0.01 mg/mL, 0.17 ± 0.01 mg/mL and 13 %± 0.01% (V/V) for ascorbic acid MSAE and MSEO, respectively.

The high antioxidant activity of the MSAE is believed to be attributed to its composition, which is rich in phenolic and flavonoid compounds with radical scavenging activity. This observation is supported by the docking analysis results, which indicate that the structure of these compounds plays a crucial role in their antioxidant properties. The antioxidant properties of plant extracts, such as MSAE, are influenced by factors including the growth environment and plant species. The soil composition, climate, and weather conditions in the growth environment affect the synthesis of phenolic and flavonoid compounds (Barzalona and Casanova, 2008). Plant species exhibit genetic variations in producing antioxidants, and the metabolic pathways involved differ among species (Barzalona and Casanova, 2008). The plant part used for extraction, extraction methods, and the maturity of the plant also contribute to variations in antioxidant content. Understanding these factors is crucial for optimizing conditions and obtaining plant extracts with desired antioxidant activities.

### Antimicrobial activity

The antimicrobial activity of MSEO and MSAE was tested against various microorganisms, and the results are summarized in Table 3. In the diffusion test, the oil that was diluted with 50% DMSO showed a strong fungicidal and fungal effect against mold fungi, with significant inhibition diameters (ID) compared to the cycloheximide control. Only three strains, *A. Niger* ID = 65 mm, *P. digitatum* ID = 53 mm, and *G. candidum* ID = 42 mm, had their minimum concentration values (MIC and MFC) calculated due to their significant results. The MIC and MFC values were 0.09% and 0.2% for *A. Niger*, 0.1% and 0.5% for *P. digitatum*, and 1% and 2% for *G. candidum*. Additionally, the yeasts *C. albicans*, *R. glutinis*, and *S. cerevisiae* had ID values of 20 mm, 13 mm, and 11 mm, respectively, indicating high sensitivity to the inhibitory effect of MSEO at a 50% dilution. However, bacteria were more resistant than fungi, with ID values of 14 mm and 15 mm for *E. coli* and *P. aeruginosa*, respectively, and ID values of 17 mm and 18 mm for *L. innocua* and *S. aureus*, respectively. Finally, it was found that the MSAE had no antimicrobial activity against any of the tested strains.

**TABLE 3:** Antimicrobial test results of MSEO and MSAE.

Microbial strains		Inhibition diameter (mm)			Minimal concentration values %	
	MSEO	MSAE	W+	W^−^	MIC	MFC
*E. coli* (ATCC 10536)	14.0 ± 1.0^aABC^	nd	20.33 ± 0.58^b^	nd	_	_
*P. aeruginosa* (ATCC 15442)	15.0 ± 1.0^aBCD^	nd	20.0 ± 1.0^b^	nd	_	_
L. innocua (ATCC 49.19)	17.0 ± 1.0^aCDE^	nd	23.67 ± 0.58^b^	nd	_	_
*S. aureus* (ATCC 6538)	18.0 ± 1.0^aDE^	nd	25.33 ± 0.58^b^	nd	_	_
*A. Niger*	65.33 ± 0.58^aH^	nd	30.33 ± 0.58^b^	nd	0.09 ± 0.00^a^	0.2 ± 0.00^a^
*P. digitatum* P22	53.67 ± 1.53^aG^	nd	31.0 ± 1.0^b^	nd	0.1 ± 0.00^a^	0.5 ± 0.00^b^
*G.candidum*	42.0 ± 1.0^aF^	nd	29.67 ± 0.58^b^	nd	1 ± 0.00b	2 ± 0.00^c^
*C. albicans*	20.67 ± 3.06^aCDE^	nd	27.67 ± 0.58^a^	nd	_	_
*R.glutinis* UMP22	13.0 ± 1.0^aAB^	nd	30.33 ± 0.58^b^	nd	_	_
*S. cerevisiae*	11.0 ± 1.0^aABCD^	nd	30.67 ± 0.58^b^	nd	_	_

MSEO: M. subtomentella Essential Oil, MSAE: M. subtomentella aqueous extract, nd: non defined, W+: Positive control (Cycloheximide for fungi and gentamicin for bacteria) W^−^: negative control (DMSO), MIC: the minimum inhibitory concentration, MFC: the minimum fungicidal concentration.

Research on novel products to control pathogenic microorganisms is an exciting area of research. Natural compounds produced by secondary plant metabolism, like essential oils, are a potentially important source of new types of biocides. In our study, MSEO and MSAE were tested against ten microbial strains. The results of this test indicated that MSEO has an antimicrobial effect on all strains tested. It has a fungicidal effect against the three molds tested, specifying against *A. Niger* with a MIC of 0.09%. Our results agree with the results found by (Mahboubi and Ghasem, 2008). These authors showed that the oil of southern Iran has a significant effect against *A. Niger* but with a higher MIC, which is of the order of 0.25% compared to our results. We also showed that Gram-positive bacteria (L. innocua ATCC 49.19 and *S. aureus* ATTC 6538) were slightly more sensitive than Gram-negative bacteria (*E. coli* ATCC 10536, *P. aeruginosa* ATCC 15442) which is in correlation with those of previous studies (Tohma et al., 2016; Köksal et al., 2017; Durmaz et al., 2022; Karagecili et al., 2023; Yılmaz et al., 2023). The reason for this variation in sensitivity could be attributed to the presence of hydrophobic lipopolysaccharides in the outer membrane of Gram-negative bacteria, which provides a protective barrier against different agents (Paracini et al., 2022). Variations in antimicrobial production between plant species are influenced by genetic variations and the different metabolic pathways involved (Barzalona and Casanova, 2008). The choice of plant part to extract, extraction methods and plant maturity also contribute to variations in antimicrobial content (Gonelimali et al., 2018; Bitwell et al., 2023). The interactions and synergies of multiple bioactive compounds contribute to the overall efficacy of plant extracts and understanding these factors is essential for optimizing conditions and obtaining plant extracts with the desired antimicrobial properties.

## Results of the molecular docking analysis

As per a 2016 report by the Tufts Center for the Study of Drug Development, the costs associated with developing and bringing a new drug to market have risen by nearly 145% in the past decade (DiMasi, Grabowski, and Hansen, 2015). Moreover, while the time required to advance a drug to clinical trials has decreased, the approval rate by the US Food and Drug Administration (FDA) has dropped to 12% (Missoum, 2020; Chraibi et al., 2021). Computer-aided drug design (CADD), which includes methods such as molecular docking and virtual screening, has been instrumental in reducing both the costs and the time involved in the drug discovery process (Surabhi and Singh, 2018). These approaches effectively steer experimental research toward optimal compounds, offering a valuable alternative to the labor-intensive and expensive high-throughput screening process (Surabhi and Singh, 2018). To corroborate the experimental data and gain insights into the potential mechanism underlying the antioxidant, antibacterial, and antifungal activities, molecular docking investigations were conducted with Glutathione reductase (PDB ID: 3GRS) (Rashid et al., 2023a), dihydrofolate reductase (PDB ID: 4M6J) (Khatun et al., 2021), and Cytochrome P450 alpha-sterol demethylase (PDB ID: 1EA1) (Taibi et al., 2023). These proteins are renowned for their roles in the antioxidant, antibacterial, and antifungal mechanisms. The listed binding energies of the formed complex between identified MSEO, and the aqueous extract’s constituents and the later proteins targets are presented in (Table 4).

**TABLE 4:** Molecular docking scores or binding affinity (kcal/mol) retrieved from *in silico* interactions of the identified compounds in MSEO, and MSAE.

N°	Compounds	3GRS (Antioxidant)	4M6J	1EA1
			(Antibacterial)	(Antifungal)
		Free Binding energy (Kcal/mol) *		
-	Native Ligand	−6.3	−7	−5.8
MSEO				
1	α-Pinene	−5.2	−5.7	−4.2
2	β-Pinene	−5	−5.7	−4.1
3	3-Octanol	−4.2	−4.7	−3.3
4	D-Limonene	−5.3	−5.9	−4.4
5	p-Mentha-3,8-diene	−5.3	−6.1	−4.1
6	9-Ethylbicyclo (3.3.1)nonan-9-ol	−5.4	−5.8	−4.8
7	(E)-3,3-Dimethyl-.delta.1,.alpha.-cyclohexaneacetaldehyde	−5.4	−5.8	−4
8	p-Menth-4 (8)-en-3-one	−5.7	−6.7	−4.7
9	Verbenone	−5.4	−5.9	−4.6
10	Caryophyllene	−6.7	−7.9	−5.8
11	1,4,7,-Cycloundecatriene	−6.3	−8	−5.1
MSAE				
1′	Salvianolic acid H	−8.5	−8.9	−8
2′	Caffeoyl hexosylpentoside	−8	−8.1	−6
3′	Rosmarinic acid	−7.4	−7.6	−6.1
4′	Feruloyl hexosylpentoside	−7.9	−7.6	−5.7

MSEO: mentha subtomentella essential oil, MSAE: mentha subtomentella aqueous extract.

### Antioxidant activity *in silico*

**FIGURE 4:**
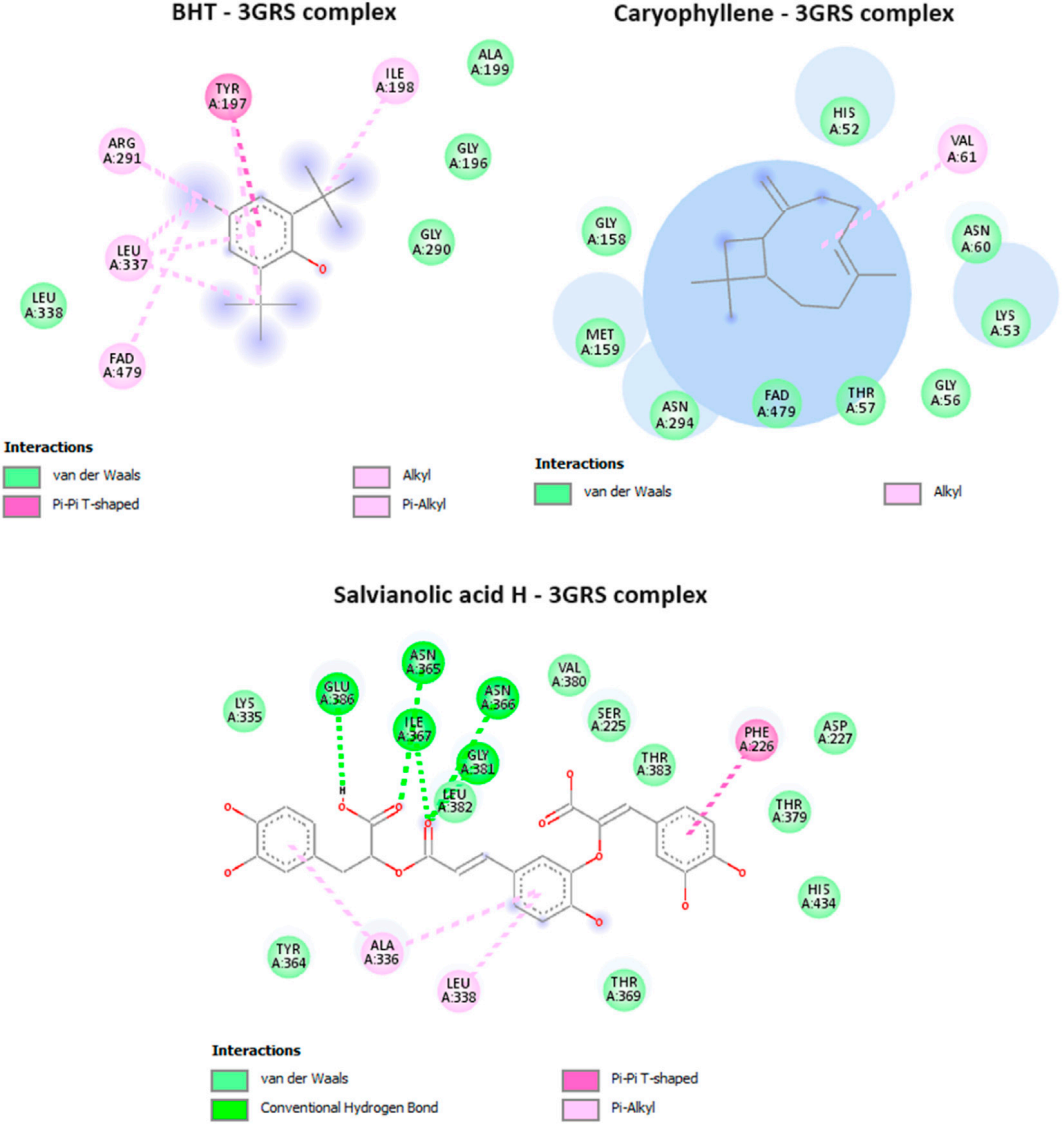
Interactions of BHT (native ligand), Caryophyllene, and Salvianolic acid H, with Glutathione reductase enzyme (PDB ID: 3GRS).

Glutathione reductase enzyme is associated with the regulation, modulation, and maintenance of redox homeostasis and oxidative stress (Couto et al., 2016). Glutathione is an essential antioxidant present in cells, and it helps protect cells from oxidative damage caused by reactive oxygen species (ROS) and free radicals. These harmful molecules can damage cell components, such as proteins, lipids, and DNA. Glutathione exists in both reduced (GSH) and oxidized (GSSG) forms. Glutathione reductase, along with other antioxidant enzymes and molecules, contributes to maintaining the cellular redox balance (Couto et al., 2016). Only two compounds from MSEO were found to exert good binding affinity (−6.3 to −6.7 kcal/mol; Table 1) towards the glutathione reductase enzyme compared with the standard antioxidant drug butylated hydroxytoluene (BHT) (−6.3 kcal/mol). While all the compounds from the MSAE were having a good inhibitory potential against GSH enzyme, ranging from −7.4 to −8.5 kcal/mol. Caryophyllene was the potent compound from MSEO, with a binding affinity of −6.7 kcal/mol, and Salvianolic acid H from MSAE, with −8.5 kcal/mol. The active binding sites of the glutathione reductase enzyme while interacting with the isolated compounds are summarized in Figure 4. A total of 13 hydrophobic interactions were noticed during the molecular docking of Caryophyllene, where the sum number of alkyl and pi-alkyl interaction was four. Salvianolic acid H, was found to exhibit 5 hydrogen bonds with the amino acid residues from the active site, which explains the potent inhibitory potential of this compound in complex with glutathione reductase enzyme.

### Antibacterial activity *in silico*

**FIGURE 5:**
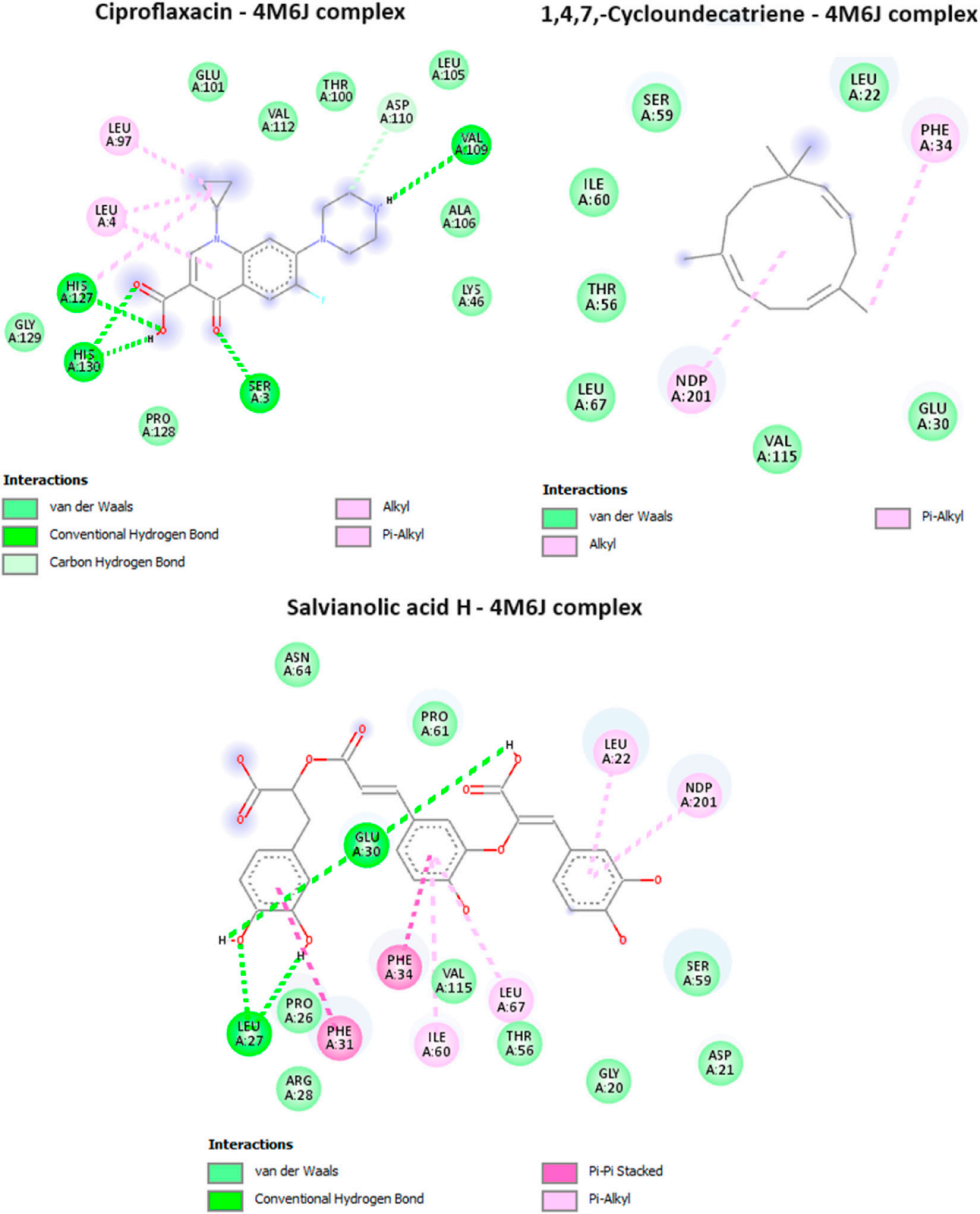
Interactions of ciproflaxacin (reference drug), 1,4,7, -Cycloundecatriene, and Salvianolic acid H, with Dihydrofolate reductase (PDB ID: 4M6J).

Dihydrofolate reductase (DHFR) is an enzyme that plays a crucial role in the biosynthesis of DNA, RNA, and amino acids by catalyzing the conversion of dihydrofolate into tetrahydrofolate. DHFR is the target of several antibiotics, most notably sulfonamides and trimethoprim. The inhibition of DHFR means the inhibition of folate synthesis, as bacteria, unlike humans, are unable to take up preformed folate from their environment and rely on *de novo* folate synthesis. The inhibition of DHFR leads to the depletion of folate and ultimately interfering with the growth and reproduction of bacterial cells. In the current study, it was found that caryophyllene, and 1,4,7, -Cycloundecatriene were having the lowest binding affinity values of −7.9, and −8 kcal/mol, in comparison with the antibacterial drug, ciprofloxacin (−7 kcal/mol) (Table 4.), showcasing the potential of these two compounds from MSEO in disrupting DHFR’s functions. All the compounds identified in the MSAE were found to have an important inhibitory potential, with values ranging from −7.6 to −8.9 kcal/mol, with Salvianolic acid H having the highest binding affinity (the lowest binding value of −8.9 kcal/mol). Salvianolic acid H exhibit two hydrogen bonds with two amino acid residues, namely, Leu A:27, and Glu A:30, from the active site (Figure 5).

### Antifungal activity *in silico*

**FIGURE 6:**
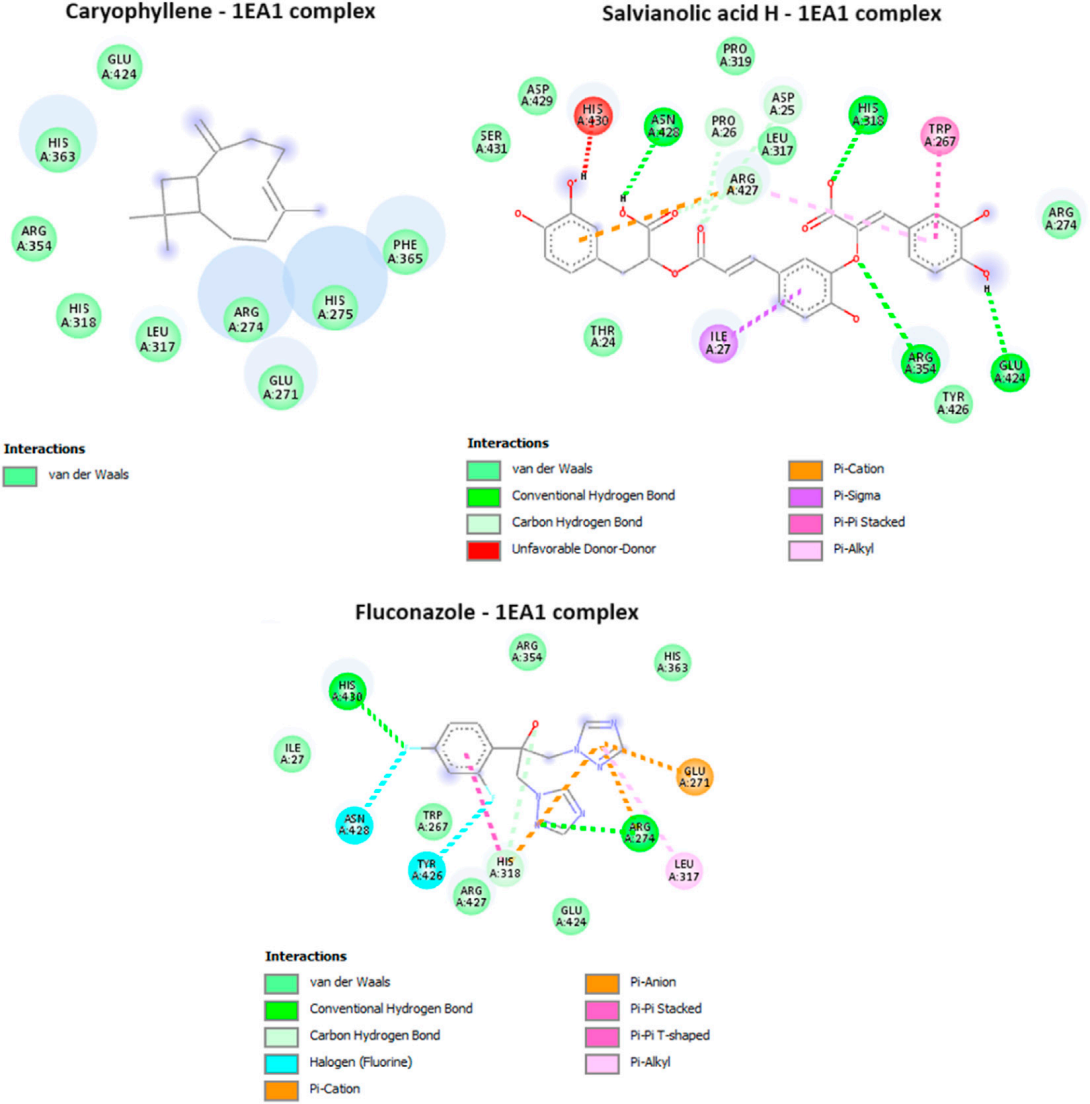
Interactions of Caryophyllene, Salvianolic acid H, and ciproflaxacin (reference drug) with Cytochrome P450 alpha-sterol demethylase (PDB ID: 1EA1).

Cytochrome P450 α-Sterol Demethylase, also known as CYP51s, occupies a pivotal position in the intricate process of sterol biosynthesis within fungal organisms. Its primary function lies in catalyzing the transformation of precursor molecules during the formation of ergosterol, a critical component in fungal cell membranes. Given its indispensable role in sterol generation (Trzaskos et al., 1986), the enzyme CYP51s has garnered substantial attention as a prime candidate for the development of antifungal medications (Sheehan et al., 1999). In our study, one compound from the essential oil was found to have a potent inhibitory potential against cytochrome P450 α-Sterol Demethylase, with a docking score of −5.8 kcal/mol, equivalent of that of the reference drug, fluconazole. For the aqueous extract only three out of the four identified compounds were having a docking score values less (ranging from −6 to −8 kcal/mol) than that of the native ligand, which suggest their potent inhibitory activity. The interactions of the potent compounds are depicted in Figure 6.

## Materials and methods

### Plant material

#### Samples preparation

The aerial parts of M. subtomentella, were collected from the Taourirt province’s eastern region in August 2021. The plant material was completely dried at 40°C, and then 100 g of the plant was boiled in 1 L of distilled water for 20 min using the decoction method to obtain the aqueous plant extract (MSAE). The plant powder underwent filtration, and the resulting mixture was subjected to dehydration at 40°C to achieve the desired form. This low-temperature drying method, commonly employed, is known for preserving volatile and heat-sensitive compounds in the sample during extract preparation, highlighting the significance of temperature control in the process (Chen et al., 2021).

We utilized the same plant to extract the essential oil from M. subtomentella (MSEO) using the steam distillation method in collaboration with the “BenLahcen” cooperative in order to obtain a sufficient yield for all our analyses.

#### GC/MS analysis

Qualitative and quantitative analysis of MSEO, the analysis was performed using gas chromatography (Shimadzu GC-2010) equipped with a fused-silica capillary column (5% phenyl methyl siloxane, 30, 0.25 mm, 0.25 µm film thickness) coupled with a mass spectrometer detector (GC-MS-QP2010). The helium as a carrier gas was adjusted to a constant pressure of 100 KPa. The oven temperature was set initially at 50°C (maintained for 1 min) followed by a gradient of 10 °C/min up to 250°C (maintained for 1 min). The injector, transfer line, and ion source temperature were set at 250°C, 250°C, and 200°C, respectively. For the qualitative and quantitative analysis (Table 1), solutions containing 1 µL of the sample diluted in hexane (50 mg/g) were injected in split mode (split ratio 50–80), and the GC-MS System was operated in scan mode. Mass spectra were recorded at 70 Ev (electron impact ionization mode) with an M/Z range of 40–3,550 a.m.u (rate and solvent delays were 5s/scan and 4.5 min, respectively). The essential oil constituents were identified by comparing their MS data with those stored in the National Institute of Standards and Technology 5NIST147) computer library. LabSolutions (version 2.5) was used for data collection and processing (Table 1).

#### HPLC-DAD identification of phenolic compounds

Chromatographic analyses were performed using an agilent 1,260 Infinity II high-performance liquid chromatography system equipped with a diode array detector, employing an eclipse XDB-C18 column (3.5 um particle size, 150 4.6 mm internal diameter, USA). Elution solvent consisted of water (A) and acetonitrile (B) with formic acid (1%, v/v) in water. The separation process was performed using an initial solvent of 2%–12.5% B during a period ranging from 0 to 6 min, increased to 12.5%–30% (B) within 23 to 23 min, and subsequently ramped to 45%–75% (B) during 33–38 min, increased to 75% B during 38–42 min, followed by 75%–100% (B), (42–47 min), 100% B (47–49 min), followed by a decreased period of 100%–2% B (49–50 min), and 2% B (50–51 min). The flow rate was set to 0.6 mL/min, and the injection volume was 10 µL. The separation of extracts was monitored using wavelengths of 254,280,300, and 340 nm. Consequently, each compound’s UV/visible spectra were measured within the 190–800 nm range.

#### Antimicrobial activity

In order to evaluate the antimicrobial activity of MSEO and MSAE against ten target strains, the agar diffusion method was used as a preliminary test. The activity was determined using the well diffusion method, following the protocol described (Brahmi et al., 2021; Brahmi et al., 2023). The microbial strains were diluted (fungi to a concentration of 10^6^ spores/mL, and bacteria to a concentration of 10^6^ CFU/mL, corresponding to 0.5 McFarland), and 100 µL of each strain were then inoculated on the surface of Potato dextrose agar (PDA) medium for fungi and Muller-Hinton Agar (MHA) medium for bacteria. Wells of 6 mm diameter were created on the culture medium, and they were then individually filled with 60 µL of each 50% dimethylsulfoxide (DMSO) (V/V) diluted MSEO and MSAE with concentrations of (5 mg/mL). The Petri dishes were kept at 4°C for 2 h to allow the diffusion of essential oils, then incubated at 25°C for fungi and 37°C for bacteria for 18 h. The diameters of the resulting inhibition zones (mm) were measured, including the diameter of the well (6 mm). The test used gentamicin (1 mg/mL) positive control for bacteria, cycloheximide (1 mg/mL) for fungi, and DMSO as a negative control.

#### Minimal concentration values (MIC and MFC)

The micro-dilution technique, using resazurin to indicate microbial growth, was employed to determine the minimum inhibitory concentration (MIC) values in this study (Brahmi et al., 2023). Resazurin is an oxy-reduction indicator used to evaluate cell growth. It is a non-fluorescent purple/blue dye that turns pink and fluorescent when oxidoreductase enzymes reduce it to resorufin in viable cells. Dilution series ranging from 0.01% to 50% were prepared in Sabouraud broth (SD). After being agitated, 180 μL of each concentration was added to the wells from a 96-well microdilution plate. Prepared microbial suspensions were diluted to a concentration of 106 spores/mL, then 20 μL was added to each well, and all tests were performed in triplicate. In this study, DMSO was used as the negative control, and Cycloheximide was used as the positive control with 1 mg/mL in the presence of fungal strains. The microdilution plates were incubated at 25°C for 18–24 h. To reveal the MIC, 5 μL of the resazurin solution at 0.01 (W/V) was added to the microbial cultures. Aliquots of 10 μL from wells without any change in color, indicating absence of growth, are transferred and seeded onto PDA agar. The agar plates are then incubated at 25°C for 18 h. The minimum fungicidal concentration (MFC) is determined as the lowest concentration without any subculture.

#### The microbial strains

Microbial strains used in this work were obtained from the Laboratory of Bioresources, Biotechnology, Ethnopharmacology and Health, Faculty of Science, UMP, Oujda. The ten microorganisms tested included four bacterial strains, namely, *Escherichia Coli* (ATCC 10536) (*E. Coli*), *P. aeruginosa* (ATCC 15442) (*P. aeruginosa*), *Listeria* innocua (ATCC 49.19) (L. innocua), *Staphylococcus aureus* (ATCC6538) (*S. aureus*), and six fungi, namely, Aspergillus Niger (*A. Niger*), *Penicillium digitatum* P22 (*P. digitatum*), *Geotrichum candidum* (*G. candidum*), *Candida albicans* (*C. albicans*), *Rhodotorula glutinis* UMP22 (*R. glutinis*), and *Saccharomyces cerevisiae* (*S. cerevisiae*).

#### DPPH scavenging assay

The test was evaluated using the *in vitro* DPPH assay, as described in (Taslimi and Gulçin, 2018; Cetin Cakmak and Gülçin, 2019; Gulcin, 2020; Gulcin and Alwasel, 2023) with minor adjustments. To determine the antioxidant properties, the substance’s MSAE and MSEO were diluted in a series of concentrations 1 to 0.02 mg/mL, 4%–50% (V/V), respectively. Then, 250 μL of each dilution was combined with 2.75 mL of a 0.1 mM methanolic DPPH solution. The mixture was incubated in darkness for 30 min, measuring 517 nm. Each experiment was repeated thrice, and the scavenging activity was calculated using the provided formula.

$$\text{Radical Scavenging Activity }\left(\%\right)=\left(\frac{A0-A1}{A0}\right)\times 100$$

A0 corresponds to the absorbance when the sample is not present, and A1 represents the absorbance when the sample is added.

Inhibitory concentration of 50% (IC_50_) of the extract was determined. Ascorbic acid in different amounts was used to determine antioxidant activity as a positive control, as mentioned in the description of the antioxidant activity of the extract.

#### Molecular docking protocol

The molecular docking analysis followed the procedure outlined in Reference (Couto et al., 2016; Khatun et al., 2021; Taibi et al., 2023). Crystallographic 3D structures of the antioxidant protein, Glutathione reductase (PDB ID: 3GRS) (Rashid et al., 2023b), the antibacterial protein, dihydrofolate reductase (PDB ID: 4M6J) (Khatun et al., 2021), and the antifungal protein, Cytochtome P450 alpha-sterol demethylase (PDB ID: 1EA1) (Taibi et al., 2023), were obtained from the RCSB Protein Data Bank (PDB) and were used as the docking targets. Autodock Tools (version 1.5.6) was employed to prepare these protein structures, involving the removal of water molecules, metal atoms, co-crystallized ligands, and other non-covalently bound substances. Kollman charges, polar hydrogens, and nonpolar hydrogens were added, and the target files were saved in the appropriate pdbqt format. The ligands derived from MSEO, and MSAE, were generated as follows: a sdf (3D conformer) file was downloaded from PubChem (https://pubchem.ncbi.nlm.nih.gov/) on 5 March 2022, and subsequently converted to a pdb file using PyMol. The final pdbqt files for the ligands were created using Autodock Tools (version 1.5.6). The docking process involved rigid molecular docking with Autodock Vina, utilizing its integrated scoring function (Trott and Olson, 2010). The dimensions of the grid box, representing the search space for docking, were adjusted to align with the active binding site, and Supplementary Table S1 provides the coordinates of this grid box. The results of the docked ligand complexes were presented in terms of ∆G binding energy values (kcal/mol). For the examination of protein–ligand binding interactions and the creation of 2D representations of molecular interactions, Discovery Studio 4.1 (Dassault Systems Biovia, San Diego, CA, USA) was employed (Kandsi et al., 2022).

#### Statistical analysis

Microbial growth results were subjected to variance analysis (ANOVA). Tukey HSD intervals were represented to compare species and treatment, with significant values at *p* < 0.05. The data analysis was performed by SPSS version 20.

## Conclusion

In conclusion, both the essential oil and aqueous extract extracted from Moroccan M. subtomentella exhibit substantial potential as viable substitutes for synthetic additives due to their unique qualities. The essential oil demonstrates robust antimicrobial capabilities, while the aqueous extract showcases impressive antioxidant properties. Notably, the presence of caryophyllene in the essential oil and the identification of salvianolic acid H as a noteworthy inhibitor of bioactive compounds through *in silico* assays align with experimental findings. These discoveries have far-reaching implications for applications in both the food and pharmaceutical sectors.

## Data Availability

The raw data supporting the conclusions of this article will be made available by the authors, without undue reservation.
